# Robotic radical prostatectomy in kidney transplant recipients: A propensity‐matched cohort study

**DOI:** 10.1002/bco2.70128

**Published:** 2025-12-18

**Authors:** Jennifer M. Slota, Rose E. Darcy, Kathryn Fink, Ridwan Alam, Nicole Handa, Sai Kumar, Clayton Neill, Yutai Li, Hiten D. Patel, Kent T. Perry, Vinayak Rohan, Satish Nadig, Ashley E. Ross, Dylan Isaacson

**Affiliations:** ^1^ Department of Urology Northwestern University Feinberg School of Medicine Chicago Illinois USA; ^2^ Department of Preventative Medicine Northwestern University Feinberg School of Medicine Chicago Illinois USA; ^3^ Division of Organ Transplantation, Department of Surgery Northwestern University Feinberg School of Medicine Chicago Illinois USA; ^4^ Transplant Center, Department of Surgery, Digestive Disease Institute Cleveland Clinic Foundation Cleveland Ohio USA

**Keywords:** kidney transplant, organ allocation, prostate cancer, robotic‐assisted laparoscopic prostatectomy, robotic‐assisted radical prostatectomy, surgical outcomes

## Abstract

**Introduction:**

We aim to evaluate the perioperative, oncologic, and survival outcomes of RARP in kidney transplant recipients (KTRs) and compare to propensity‐matched controls.

**Patients and Methods:**

This was a single‐institution retrospective cohort study using the Northwestern Enterprise Data Warehouse. We identified eight KTRs who underwent RARP from January 2018–September 2024 and propensity matched them 1:4 with non‐KTR controls using age, body mass index, and pathologic Gleason score. Outcomes were assessed using Wilcoxon rank sum and Fisher's exact tests. Overall survival was analysed using Kaplan–Meier and univariable Cox proportional hazards models.

**Results:**

All RARPs in KTRs were completed robotically. Median time from kidney transplant to RARP was 11.1 years (8.9–15.1). KTRs had higher Charlson Comorbidity Index (9.5 vs 4; *p* < 0.001) but similar operative time (198.5 vs 201; *p* = 0.8), estimated blood loss (125 vs 90 ml; *p* = 0.7), and length of hospital stay (1 midnight in both; *p* = 0.2). KTRs experienced no major complications, graft injuries, episodes of acute kidney injury, or 90‐day readmissions. The 30‐day urinary tract infection rate was higher in KTRs (25% vs 0%; *p* = 0.036), who had a median catheterization duration of 11 days (8–12.5). Surgical margin positivity (29% vs 19%, *p* = 0.6) and biochemical recurrence rates (13% vs 6.3%, *p* = 0.5) did not differ. Median follow‐up time was 3.2 years in KTRs vs 1.7 years in controls (*p* = 0.13). Allograft function remained stable at 12 months. One KTR died from renal failure 44 months after RARP; none developed metastases or died of PCa.

**Conclusion:**

RARP in kidney transplant recipients is feasible and safe for experienced surgeons, with comparable surgical and oncologic outcomes as compared to matched controls. Higher UTI rates suggest modified catheter removal strategies could be considered.

## 1 INTRODUCTION

Kidney transplantation (KT) is the most definitive treatment for end‐stage renal disease, conferring improved survival and quality of life compared to dialysis.^1^ As transplant longevity increases, kidney transplant recipients (KTRs) increasingly face age‐related malignancies, with genitourinary cancers—particularly prostate cancer (PCa)—being among the most common.^2^

Managing localized PCa in KTRs is complex. Although active surveillance, radiotherapy, and surgery are viable options in the general population, radiotherapy is often avoided in KTRs due to risks to the allograft, including ureteral stricture and radiation nephropathy.^3^ Consequently, radical prostatectomy (RP) remains the preferred curative option. Robotic‐assisted radical prostatectomy (RARP), now the dominant surgical approach for PCa, offers favourable perioperative outcomes due to its minimally invasive nature but requires technical adaptations in KTRs due to long‐term immunosuppression, proximity to the graft and changes in surgical planes.^4^ Chronic immunosuppression further complicates postoperative management by increasing infection risk and impairing wound healing.^5^

Although the existing literature suggests that RARP is a reasonable approach to PCa in KTRs, comparative studies have been limited.^6^ Here we compare perioperative, oncologic, and allograft outcomes following RARP in KTRs as compared to non‐KTRs within a large academic health system database. We aim to further inform operative planning and postoperative management in this growing population.

## 2 PATIENTS AND METHODS

### 2.1 Study design and population

We conducted a retrospective cohort study of adult men undergoing RARP for localized PCa between January 1, 2018 and September 30, 2024 at a single academic health system. Of 4122 eligible patients, 8 KTRs were identified and matched 1:4 to non‐KTR controls using propensity scores based on age, body mass index (BMI), and final Gleason grade.

### 2.2 Data collection

Clinical, demographic and oncologic data were obtained from the Northwestern Medicine Enterprise Data Warehouse and validated by manual chart review. Baseline variables included age, self‐identified race and ethnicity, BMI, Charlson Comorbidity Index (CCI), haemoglobin, serum prostate‐specific antigen (PSA), magnetic resonance imaging (MRI) findings, National Comprehensive Cancer Network (NCCN) risk classification, and biopsy Gleason grade (GG). Perioperative variables included estimated operative time, blood loss (EBL), length of stay (LOS), surgeon volume (high‐volume defined as > 25 RARPs/year), and readmissions. Undetectable PSA was defined as <0.02 ng/ml; biochemical recurrence (BCR) as PSA ≥ 0.2 ng/ml on two consecutive tests. Renal function was assessed by serum creatinine and estimated glomerular filtration rate (eGFR).

KTR‐specific data included surgical approach, graft laterality, graft function, KT‐to‐RARP interval, lymphadenectomy technique, and duration of postoperative catheterization. Complications were graded via Clavien‐Dindo classification.^7^ The Memorial Sloan Kettering Cancer Center (MSKCC) pre‐RP nomogram estimated lymph node involvement (LNI) probability.^8^

### 2.3 Statistical analyses

Propensity matching used logistic regression and nearest neighbour methods (callipers of 5 for age and 2 for BMI). Balance was assessed using standardized mean differences. Descriptive statistics were compared using Wilcoxon rank‐sum and Fisher's exact tests.

Kaplan–Meier methods estimated overall survival (OS), with log‐rank tests for comparison. Univariable Cox regression and inverse probability of treatment weighting were used to assess mortality risk in the matched and unmatched groups (Table 6, Supplementary Table 4). Follow‐up time was included in survival analyses to account for differing median durations between cohorts. Analyses used R (v4.2.0, R Foundation, Vienna, Austria); significance was set at *p* < 0.05.

### 2.4 Surgical modifications

**FIGURE 1:**
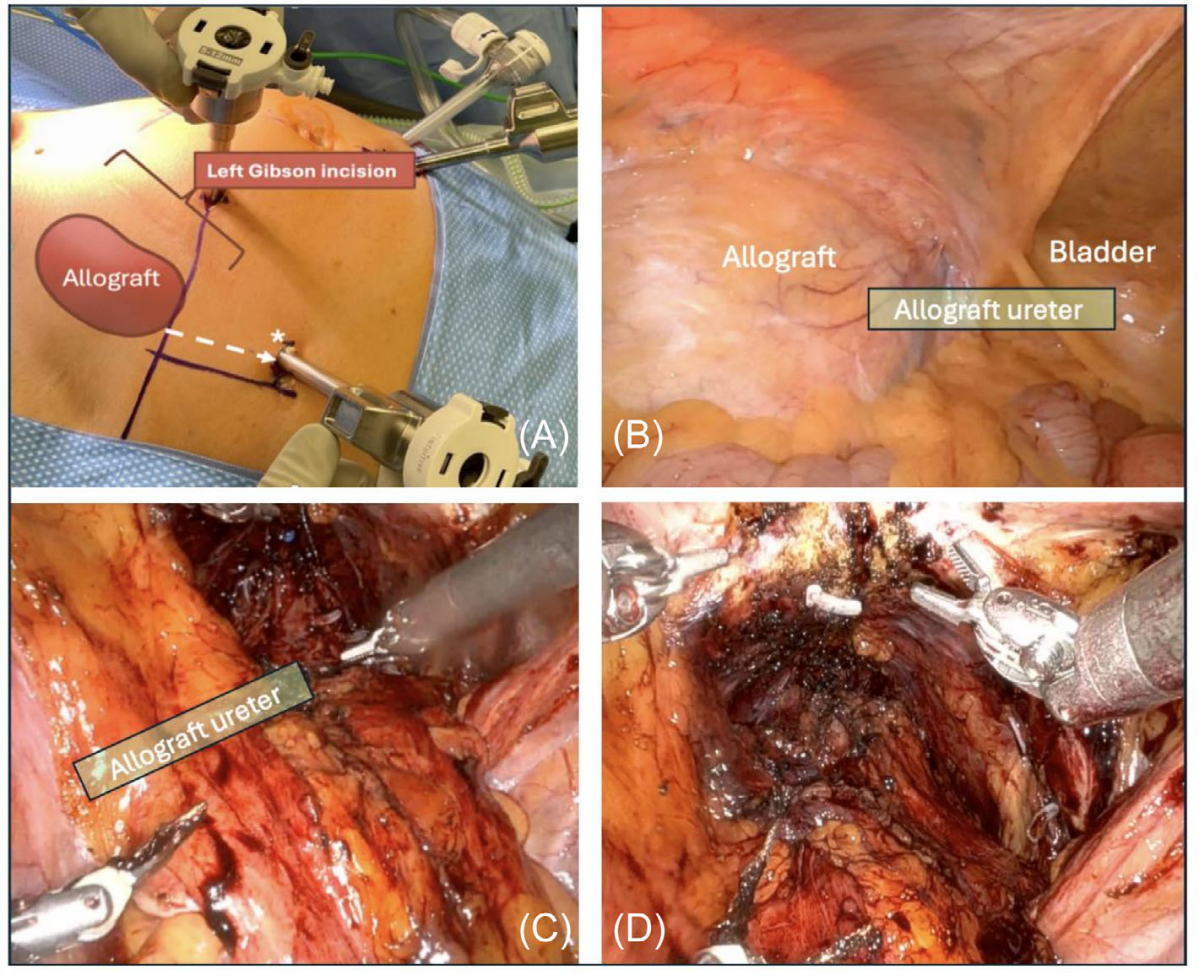
Title: intraoperative findings during RARP in kidney transplant recipient. Caption: intraoperative images showing a) adjustment of the left lateral robotic port cranially to avoid the allograft, estimated using the left‐sided Gibson incision, b) the transplant kidney in the left hemi‐pelvis lateral to the bladder with approximate location of the transplant ureter, c) bladder release after prostate removal was more extensive on the right than the left side, d) pelvic lymph node dissection performed on the right but omitted on the left side.

Standard RARP utilized four robotic and two assistant ports, as previously described.^9^ Port configuration typically does not require modification; however, in 2 of 8 KTRs (25%), the most lateral port ipsilateral to the allograft was shifted cranially to avoid impingement on the graft by the robotic arms while working in the pelvis (Figure 1a).

Two RARP techniques are employed at our institution based on surgeon preference: a standard transperitoneal (anterior) approach involving dissection of the space of Retzius, and a Retzius‐sparing (posterior) approach. The Retzius‐sparing technique obviates entry into the extraperitoneal space, thereby minimizing contact with the renal graft, but its technical demands have been well‐documented.^10^ For anterior approaches, dissection was minimized on the graft side to reduce the risk of inadvertent ureteral/vascular injury. In such cases, the bladder may not be fully mobilized on the graft side (Figure 1c). Furthermore, understanding common techniques in renal transplantation, particularly the anterolateral location of the ureterovesical anastomosis, is critical when dissecting this space (Figure 1b). The transplant ureter was not routinely stented but could be if an injury were suspected. Pelvic lymph node dissection (PLND) was universally omitted on the graft side due to the proximity to the vascular anastomoses (Figure 1d).

### 2.5 Immunosuppression

Most KTRs (87.5%) were maintained on a combination of tacrolimus and an antimetabolite (mycophenolate mofetil or mycophenolate sodium) at the time of RARP; 2 (25%) were also taking corticosteroids. Immunosuppression regimens were unchanged perioperatively.

## 3 RESULTS

### 3.1 Cohort characteristics

Of 4122 RARP patients, 8 (0.2%) were KTRs. Median age was 58.5 years in KTRs and 64.0 in unmatched controls (Table 1). The majority of both groups were white (50% and 74%, respectively, *p* = 0.1). KTRs had higher CCI (9.5 vs 4.0; *p* < 0.001) and lower preoperative haemoglobin (11.8 vs 13.6 g/dl; *p* = 0.026). PCa characteristics were similar between groups. Median time from diagnosis to RARP was 3–4 months (*p* = 0.3).

**TABLE 1:** Baseline characteristics of RARP patients by KTR status.

	KTRs (*n* = 8)	Unmatched controls (*n* = 4114)	*p*	Matched controls (*n* = 32)	*p*	SMD (95% CI)
Age (year)	58.5 (52.5, 65.5)	64.0 (58.0, 69.0)	0.1	57.0 (53.0, 64.5)	0.7	−0.162 (−0.937, 0.614)
BMI (kg/m^2^)	26.45 (24.13, 34.84)	27.82 (25.41, 30.72)	0.9	28.59 (24.77, 31.77)	>0.9	−0.058 (−0.833, 0.717)
Race			0.1		0.2	1.03 (0.225, 1.84)
Asian	0	84 (2%)		1 (3.1%)		
Black or African American	3 (38%)	440 (11%)		3 (9.4%)		
White	4 (50%)	3030 (74%)		23 (72%)		
Other	1 (13%)	228 (5.5%)		1 (3.1%)		
Did not respond	0	332 (8.1%)		4 (13%)		
Ethnicity			0.7		0.7	0.609 (−0.178, 1.39)
Hispanic or Latino	0	176 (4.3%)		1 (3.1%)		
Not Hispanic/Latino	8 (100%)	3499 (85%)		27 (84%)		
Did not respond	0	439 (11%)		4 (13%)		
Charlson comorbidity index	9.5 (7.5, 10.5)	4.0 (4.0, 6.0)	<0.001	4.0 (3.0, 4.0)	<0.001	−2.28 (−3.20, −1.35)
30‐day pre‐op haemoglobin (g/dL)	11.8 (10.3, 13.1)	13.6 (12.8, 14.4)	0.004	13.6 (12.5, 14.3)	0.026	1.35 (0.303, 2.39)
*Missing*	*1*	*1939*		*21*		
† 30‐day pre‐op creatinine (mg/dL)	1.25 (0.96, 1.44)	1.00 (0.90, 1.14)	0.056	1.09 (0.99, 1.25)	0.4	−0.745 (−1.78, 0.299)
*Missing*	*1*	*2283*		*22*		
† 30‐day pre‐op eGFR (mL/min/1.73 m^2)	62.0 (59.0, 87.0)	65.0 (60.0, 87.0)	0.6	71.5 (60.0, 90.0)	0.6	0.314 (−7.04, 1.33)
*Missing*	*1*	*2275*		*22*		
Prostatic health index	54.6 (48.25, 70.95)	47.80 (37.80, 63.96)	0.4	54.35 (40.90, 62.45)	0.7	−0.461 (−1.50, 0.581)
*Missing*	*3*	*2913*		*19*		
MRI prostate volume (mL)	35.0 (24.0, 52.34)	39.0 (29.6, 53.0)	0.5	37.65 (27.0, 44.0)	>0.9	0.109 (−0.726, 0.944)
*Missing*	*1*	*1105*		*6*		
MRI PSA density (ng/mL/cc)	0.17 (0.14, 0.34)	0.15 (0.10, 0.24)	0.3	0.14 (0.10, 0.24)	0.2	−0.475 (−1.32, 0.367)
*Missing*	*1*	*1196*		*6*		
PSA density ≥0.15 ng/ml/cc	4 (57%)	1529 (52%)	>0.9	11 (42%)	0.7	−0.300 (−1.14, 0.538)
*Missing*	*1*	*1196*		*6*		
Initial serum PSA (ng/mL)	6.09 (5.40, 7.60)	5.94 (4.43, 8.76)	0.8	6.26 (4.02, 8.43)	0.9	−0.127 (−0.913, 0.660)
*Missing*	*0*	*388*		*3*		
MRI PIRADS score			0.5		0.5	0.883 (−0.107, 1.87)
1–2	0	71 (2.5%)		0		
3	0	373 (13%)		5 (21%)		
4	2 (40%)	1454 (51%)		12 (50%)		
5	3 (60%)	954 (33%)		7 (29%)		
*Missing*	*3*	*1262*		*8*		
Biopsy Gleason grade			0.8		0.9	0.633 (−0.187, 1.45)
1	0	222 (7.7%)		1 (4.3%)		
2	4 (50%)	1298 (45%)		13 (57%)		
3	4 (50%)	851 (29%)		7 (30%)		
4	0	296 (10%)		2 (8.7%)		
5	0	219 (7.6%)		0		
*Missing*	*0*	*1228*		*9*		
NCCN risk classification			0.4		0.6	0.796 (−0.033, 1.62)
Very low	0	15 (0.5%)		0		
Low	1 (13%)	150 (5.4%)		1 (4.3%)		
Favourable intermediate	2 (25%)	932 (34%)		10 (43%)		
Unfavourable intermediate	5 (63%)	1025 (37%)		9 (39%)		
High	0	430 (16%)		2 (8.7%)		
Very high	0	210 (7.6%)		1 (4.3%)		
*Missing*	*0*	*1352*		*9*		
Time from PCa diagnosis to RARP (month)	3.43 (3.02, 6.05)	3.09 (2.10, 5.32)	0.3	3.06 (2.27, 4.67)	0.3	−0.155 (−0.933, 0.623)
*Missing*	*0*	*10*		*1*		
* Follow‐up time (month)	38.93 (27.20, 44.30)	26.84 (10.81, 48.62)	0.3	20.45 (5.72, 40.95)	0.13	−0.656 (−1.44, 0.132)

*n* (%); median (Q1, Q3).

Abbreviations: BMI = body mass index; CI = confidence interval; eGFR = estimated glomerular filtration rate; KTR = kidney transplant recipient; MRI = magnetic resonance imaging; NCCN = National Comprehensive Cancer Network; PCa = prostate cancer; PIRADS = prostate imaging reporting and data system; PSA = prostate‐specific antigen; RARP = robotic‐assisted radical prostatectomy; SMD = standardized mean difference.

* Includes only patients with follow‐up after RARP (*n* = 8 KTR, *n* = 3997 controls).

^†^ Excludes‐dialysis dependent patients (*n* = 1 KTR, *n* = 11 controls).

**FIGURE 2:**
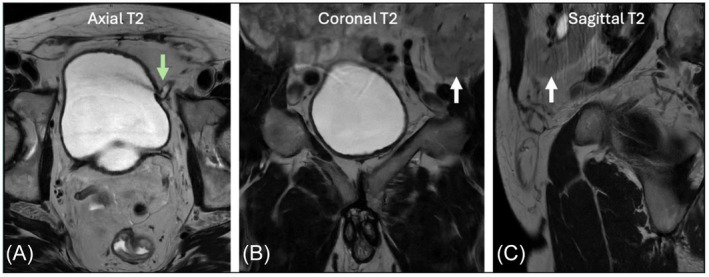
Title: prostate MRI demonstration of transplant kidney and ureter in the left iliac fossa. Caption: prostate MRI images of the same patient, showing a) the transplant ureter entering the left bladder wall at the site of implantation (green arrow), b) and c) the transplant kidney in the left hemi‐pelvis lateral to the bladder (white arrows).

Seven KTRs had their allograft located in the left hemipelvis; one had a right‐sided graft (Table 2, Figure 2). Median age at KT was 46.5 years, with an 11.1‐year median KT‐to‐RARP interval. At RARP, one KTR was dialysis‐dependent due to chronic rejection and subsequently underwent retransplantation 24.2 months after RARP. Among KTRs, median predicted LNI risk was 8%.

**TABLE 2:** KTR‐specific characteristics and perioperative parameters (*n* = 8).

Age at kidney transplant (years)	46.50 (40.49, 56.18)
Aetiology of renal failure	
FSGS	2 (25%)
IgA nephropathy	1 (13%)
Membranous glomerulonephritis	1 (13%)
Polycystic kidney disease	2 (25%)
Type 1 diabetes mellitus	1 (13%)
Type 2 diabetes mellitus	1 (13%)
Renal graft position	
Right iliac fossa	1 (13%)
Left iliac fossa	7 (88%)
eGFR 12 months after KT (mL/min/1.73 m^2^)	56.0 (51.0. 60,0)
*Missing*	*2*
Functioning graft at RARP	7 (88%)
Dialysis dependence at RARP	1 (12.5%)
Time between KT and RARP (years)	11.13 (8.85, 15.09)
MSK pre‐radical prostatectomy nomogram probability predictions	
15‐year PCa‐specific survival (%)	96.0 (91.5, 97.5)
5‐year progression‐free probability after RP (%)	67.5 (54.0, 77.0)
10‐year progression‐free probability after RP (%)	52.0 (38.0, 64.0)
Organ‐confined disease (%)	50.5 (32.5, 61.0)
Extracapsular extension (%)	48.5 (42.0, 66.0)
Lymph node involvement (%)	8.0 (6.0, 12.5)
Seminal vesicle invasion (%)	6.0 (4.5, 11.0)
Surgical approach	
Retzius‐sparing	1 (13%)
Transperitoneal	7 (88%)
High‐volume surgeon (>25 RARPs/year)	8 (100%)
RARP lymphadenectomy extent	
Contralateral only	5 (63%)
Ipsilateral only	0
Bilateral	0
None	3 (38%)
Duration of catheterization (day)	11.0 (8.0, 12.5)
AKI episode after RARP (during index surgical hospitalization)	0
* Clavien‐Dindo 30‐day complications	
Minor (grade II)	3
Major (grade ≥III)	0
† 6‐months post‐RARP creatinine (mg/dL)	1.26 (1.02, 1.85)
*Missing*	*1*
† 12‐months post‐RARP creatinine (mg/dL)	1.33 (1.09, 1.89)
*Missing*	*2*
† 6‐months post‐RARP eGFR (mL/min/1.73 m^2^)	62.0 (40.0, 80.0)
*Missing*	*1*
† 12‐months post‐RARP eGFR (mL/min/1.73 m^2^)	60.0 (39.0, 69.0)
*Missing*	*2*

*n* (%); median (Q1, Q3).

Abbreviations: AKI = acute kidney injury; eGFR = estimated glomerular filtration rate; FSGS = focal segmental glomerulonephritis; IgA = immunoglobulin A; KT = kidney transplant; KTR = kidney transpanat recipient; MSK = Memorial Sloan Kettering; PCa = prostate cancer; RARP = robotic‐assisted radical prostatectomy; RP = radical prostatectomy.

^†^ Excludes‐dialysis dependent patients (*n* = 1 KTR, *n* = 11 controls).

* Patients could be accounted for more than once.

### 3.2 Surgical and perioperative outcomes

All RARPS were completed robotically without conversions or graft injuries. A transperitoneal approach was utilized for seven KTRs; one underwent Retzius‐sparing approach. Operative time (198.5 vs 201; *p* = 0.3), EBL (125 vs 90 ml; *p* = 0.7), and LOS (1 midnight for both; *p* = 0.2) were similar between cohorts before and after matching (Table 3, Supplemental Table 1). No KTR experienced major (grade ≥III) complications, transfusions, or acute kidney injury (AKI) (Table 2) during hospitalization. PLND was omitted on graft side for all KTRs; three (38%) had no PLND. Total number of lymph nodes removed was lower in KTRs (3 vs 8 nodes, *p* = 0.01).

**TABLE 3:** Perioperative parameters of RARP patients by KTR status.

	KTRs (*n* = 8)	Matched controls (*n* = 32)	*p*	SMD (95% CI)
Operating time (min)	198.5 (146.5, 255.5)	201.0 (164.5, 244.5)	0.8	−0.266 (−1.04, 0.511)
Estimated blood loss (mL)	125 (50, 175)	90 (50, 200)	0.7	0.009 (−0.768, 0.787)
*Missing*	*0*	*1*		
Intra‐operative blood transfusion	0	0	‐	0 (−0.775, 0.775)
Post‐operative blood transfusion	0	0	‐	0 (−0.775, 0.775)
High‐volume surgeon (≥25 RARPs/year)	8 (100%)	28 (88%)	0.6	−0.535 (−1.32, 0.249)
Total lymph nodes removed at RARP	3 (0.4)	8 (4, 10)	0.01	1.31 (0.347, 2.27)
*Missing*	*2*	*9*		
Hospitalization length (days)	1 (1,2)	1 (1,1)	0.2	−0.528 (−1.36, 0.307)
*Missing*	*1*	*3*		
24‐hour post‐op haemoglobin (g/dL)	11.6 (10.4, 13.7)	12.6 (11.9, 13.3)	0.3	0.540 (−0.371, 1.45)
*Missing*	*1*	*17*		

*n* (%); median (Q1, Q3).

Abbreviations: CI = confidence interval; KTR = kidney transplant recipient; RARP = robotic‐assisted radical prostatectomy; SMD = standardized mean difference.

Two KTRs (25%) experienced minor complications (both grade II), with a total of three events: one episode of hyperkalemia and two urinary tract infections (UTIs). 30‐day UTI rate was higher in KTRs (25% vs 0%, *p* = 0.036) (Table 4). Median catheter duration in KTRs was 11 days.

**TABLE 4:** Short and long‐term outcomes of RARP patients by KTR status.

	KTRs (*n* = 8)	Matched controls (*n* = 32)	*p*	SMD (95% CI)
† 30‐day post‐op creatinine (mg/dL)	1.55 (1.23, 2.04)	1.09 (0.98, 1.22)	0.002	−1.92 (−2.98, −0.856)
*Missing*	*1*	*13*		
† 30‐day post‐op eGFR (mL/min/1.73 m^2^)	57.5 (41.0, 60.0)	60.00 (60.00, 85.00)	0.029	1.19 (0.212, 2.16)
*Missing*	*1*	*13*		
30‐day urgent care visit	1 (13%)	0	0.2	−0.535 (−1.32, 0.249)
90‐day hospital readmission	0	1 (3.1%)	>0.9	0.254 (−0.523, 1.03)
90‐day ICU admission	0	0	‐	0 (−0.775, 0.775)
30‐day urine culture	4 (50%)	9 (28%)	0.4	−0.460 (−1.24, 0.321)
30‐day urinary tract infection	2 (25%)	0	0.036	−0.816 (−1.61, −0.021)
Undetectable PSA achieved	8 (100%)	25 (78%)	>0.9	−0.490 (−1.28, 0.304)
Time to undetectable PSA (month)	4.1 (3.2, 6.3)	3.3 (2.6, 4.0)	0.2	−0.083 (−0.879, 0.713)
*Missing*	*0*	*7*		
Biochemical recurrence	1 (13%)	2 (6.3%)	0.5	−0.216 (−0.992, −0.561)
Metastasis	0	0	‐	0 (−0.775, 0.775)
Had radiation or hormonal therapy	1 (13%)	4 (13%)	>0.9	0 (−0.775, 0.775)
Overall mortality	1 (13%)	0	0.2	−0.535 (−1.32, 0.249)
Prostate cancer‐specific mortality	0	0	‐	‐
Time from RARP to death (month)	44	N/A		

*n* (%); median (Q1, Q3).

Abbreviations: CI = confidence interval; eGFR = estimated glomerular filtration rate; ICU = intensive care unit; KTR = kidney transplant recipient; PSA = prostate‐specific antigen; RARP = robotic‐assisted radical prostatectomy; SMD = standardized mean difference.

* Includes only patients with follow‐up after RARP (*n* = 8 KTR, *n* = 3997 controls).

† Excludes‐dialysis dependent patients (*n* = 1 KTR, *n* = 11 controls).

### 3.3 Oncologic outcomes

Tumour pathology was similar between matched and unmatched groups (Table 4, Supplementary Table 3). All KTRs had grade group 2 or 3 tumours on final pathology, with 75% harbouring grade group 2 (Table 5). Among 7 staged KTRs, 5 (71%) had pT3 tumours. Two (29%) had positive surgical margins (PSM; vs 19% in controls, *p* = 0.6), one of whom developed BCR and was treated with salvage radio‐ and hormonal therapy. Time to undetectable PSA (4.1 vs 3.3 months; *p* = 0.2) was similar between cohorts before and after matching (Table 4, Supplementary Table 2).

**TABLE 5:** Histopathologic and oncologic outcomes of RARP patients by KTR status.

	KTRs (*n* = 8)	Matched controls (*n* = 32)	*p*	SMD (95% CI)
Gleason grade on surgical pathology			0.7	0.272 (−0.505, 1.05)
1	0	0		
2	6 (75%)	20 (63%)		
3	2 (25%)	12 (38%)		
4	0	0		
5	0	0		
*Missing*	*0*	*0*		
Pathologic tumour stage			0.4	0.450 (−0.374, 1.27)
pT0 or pT1A	0	0		
T2	2 (29%)	16 (50%)		
T3	5 (71%)	16 (50%)		
T4	0	0		
TX	0	0		
*Missing*	*1*	*0*		
Node stage			>0.9	0.289 (−0.531, 1.11)
N0	5 (71%)	24 (75%)		
N1	0	1 (3.1%)		
NX	2 (29%)	7 (22%)		
*Missing*	*1*	*0*		
Surgical margin status			0.6	0.233 (−0.587, 1.05)
Involved (positive)	2 (29%)	6 (19%)		
Uninvolved (negative)	5 (71%)	26 (81%)		
*Missing*	*1*	*0*		

*Note: n* (%); median (Q1, Q3).

Abbreviations: CI = confidence interval; KTR = kidney transplant recipient; RARP = robotic‐assisted radical prostatectomy; SMD = standardized mean difference.

### 3.4 Graft function

Among KTRs not on dialysis, median serum creatinine increased from 1.25 to 1.55 mg/dl and eGFR declined from 62 to 57.5 ml/min/1.73m^2^ when comparing values from within 30 days preoperatively to 30 days postoperatively (Table 1, Table 4). Values returned to baseline and remained stable at 6 and 12 months.

### 3.5 Survival outcomes

**FIGURE 3:**
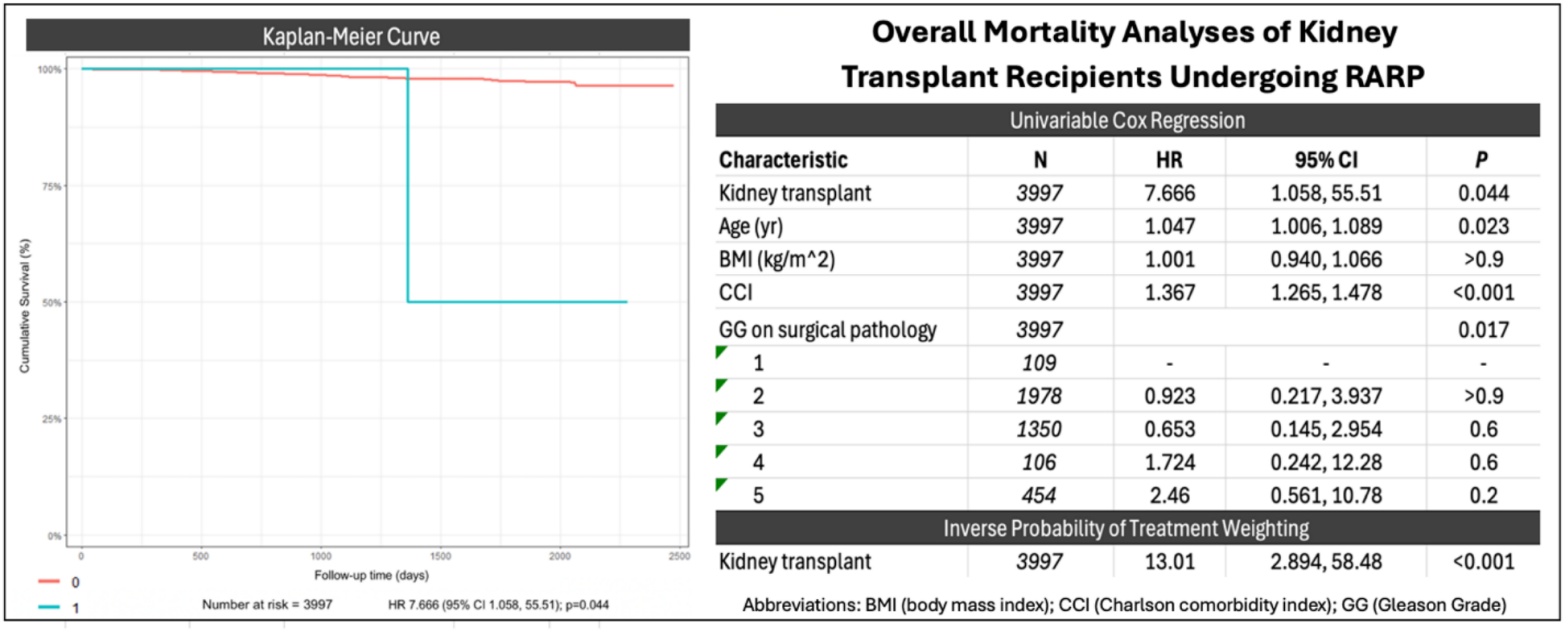
Title: overall survival of RARP patients by KTR status (also uploaded separately).

One KTR died from renal failure 44 months post‐RARP (Table 4). Overall mortality during the study period was higher in KTRs (13% vs 0%, *p* = 0.2). In unmatched analysis, KTR status was independently associated with higher mortality (hazard ratio [HR] 7.67, 95% confidence interval [CI] 1.06–55.51; *p* = 0.045) (Figure 3), confirmed in IPTW analysis (HR 13.01, 95%, CI 2.89–58.48; *p* < 0.001) (Table 6). Kaplan–Meier curves for overall survival are shown in Figure 3.

**TABLE 6:** Overall mortality analyses of unmatched cohort.

Univariable cox regression				
Characteristic	*N*	HR	95% CI	*p*
Kidney transplant	*3997*	7.666	1.058, 55.51	0.044
Age (year)	*3997*	1.047	1.006, 1.089	0.023
BMI (kg/m^2^)	*3997*	1.001	0.940, 1.066	>0.9
CCI	*3997*	1.367	1.265, 1.478	<0.001
GG on surgical pathology	*3997*			0.017
1	*109*	‐	‐	‐
2	*1978*	0.923	0.217, 3.937	>0.9
3	*1350*	0.653	0.145, 2.954	0.6
4	*106*	1.724	0.242, 12.28	0.6
5	*454*	2.46	0.561, 10.78	0.2
Inverse probability of treatment weighting				
Kidney transplant	*3997*	13.01	2.894, 58.48	<0.001

## 4 DISCUSSION

The KTR population is growing and aging due to advances in immunosuppression, improved graft longevity, and expanded eligibility. One of the fastest‐growing subgroups, adults aged ≥65 years, now comprises 24.9% of KTRs, up from 3.4% in 1990.^11^, ^12^ This demographic shift has led to a rise in age‐associated malignancies, including PCa. While immunosuppression may not substantially alter PCa risk or progression, advanced age at transplantation remains a primary driver of malignancy risk.^13^, ^14^ Despite the growing burden of PCa in KTRs, no standardized treatment guidelines exist, resulting in varying clinical practices. RP has emerged as the preferred curative option, avoiding the ureteral and graft toxicity of radiation.^4^, ^15^, ^16^ RARP, now the dominant surgical approach, offers reduced morbidity and faster recovery.^17^ Although several small series have reported the feasibility of RARP in KTRs, comparative data remain scarce. To date, only two matched cohort studies—both from France—have compared RARP outcomes in KTRs versus non‐KTRs.^3^, ^18^

In this retrospective matched cohort study, we evaluated perioperative, oncologic, and graft outcomes following RARP in KTRs compared to non‐KTRs in a large, multi‐center U.S. health system. Perioperative parameters were comparable between KTRs and non‐KTRs. All achieved an undetectable PSA, and only one experienced BCR (13%). These findings align with prior evidence, including the systematic review by Piana et al., and further support RARP as a safe and effective option in appropriately selected KTRs.^6^ Our study adds valuable insights as the first U.S.‐based matched comparison between KTRs and non‐KTR controls.

### 4.1 Surgical and perioperative outcomes

Our findings build on prior matched studies by Léonard et al. and Felber et al., which similarly reported no significant differences in operative time or blood loss between KTRs and controls.^3^, ^18^ However, both reported longer LOS for KTRs, which appear to be attributable to a higher rate of perioperative complications such as acute kidney injury and cardiopulmonary events. In contrast, LOS in our study was equivalent between groups, in part because of an absence of major complications.

In our KTR cohort, postoperative complications were limited to UTIs and transient hyperkalemia. UTIs remain the most common infectious complication in KTRs, likely exacerbated by immunosuppression and prolonged catheterization.^19^ Our significantly higher 30‐day UTI rate in KTRs (25% vs 0%, *p* = 0.036) aligns with prior data and underscores the importance of close perioperative infection surveillance. Prolonged catheterization time in KTRs (median 11 days) compared to standard, though cautious, falls within previously published ranges for KTRs (5–18 days), likely reflecting anatomical considerations and anastomotic healing concerns unique to transplant recipients.^6^, ^20^ These findings highlight the importance of strategies to mitigate perioperative infection risk, including modified peri‐catheter removal antibiotic regimens and proactive infectious surveillance.

Importantly, all KTRs in our study were managed by high‐volume RARP surgeons (>25/year)in a health system with a comprehensive transplant program, emphasizing the importance of institutional and surgeon experience in attaining favourable perioperative outcomes for KTRs undergoing RARP.^21^, ^22^

### 4.2 Pelvic lymph node dissection

Pelvic lymphadenectomy is challenging in KTRs due to proximity of the allograft, its ureter, and its vascular anastomoses. While PLND during RARP can offer important staging information and guide adjuvant therapy, its application in KTRs requires careful risk–benefit considerations. Piana et al. emphasizes the value of predictive nomograms to inform selective lymphadenectomy in this population given the variability of practices and scarcity of data on lymph nodes removed, making this an aspect of surgical decision‐making that warrants further investigation.^6^

Consistent with this risk‐adapted approach, we universally omitted ipsilateral PLND in KTRs and omitted PLND entirely in 38%. Although our KTR cohort had a median predicted LNI risk of 8%—exceeding the typical threshold for recommended PLND—no nodal metastases were identified, and nodal yield was understandably lower compared to matched controls (3 vs. 8 nodes, *p* = 0.002).^8^, ^23^, ^24^ Similar selective strategies have been reported by Le Clerc et al., Léonard et al., and Polcari et al., who modified PLND based on anatomy and risk stratification.^3^, ^18^, ^25^

While Marra et al. have demonstrated that ipsilateral PLND may be feasible using robotic magnification and meticulous dissection, they appropriately caution that graft‐threatening complications—though rare—carry disproportionate consequences.^16^ Our findings support a selective, anatomy‐informed approach to PLND can safely balance oncologic staging and graft preservation in appropriately selected KTRs. Future work should refine risk thresholds for performing PLND.

### 4.3 Oncologic outcomes

Despite a high proportion of ≥pT3a tumours in our KTR cohort, no nodal involvement, metastatic progression, or PCa‐specific mortality occurred. Our high rates of upstaging and pT3 disease mirror findings by Polcari et al., while contrasting with lower pT3a disease rates reported by Le Clerc et al. and Mistretta et al.^25^, ^26^, ^27^ However, in our analysis, pT3 disease rates did not significantly differ between KTRs and non‐KTRs (71% vs 50%, *p* = 0.4), consistent with comparative findings by Felber et al. and Léonard et al. though both reported lower overall rates of high‐risk disease (11 of 39 [28%] and 1 of 27 [3.7%], respectively).^3^, ^18^ The underlying drivers of upstaging in this population remain unclear but may reflect immunosuppression, altered tumour biology, or delayed diagnosis in KTRs. These findings raise nuanced considerations around the role of conservative management strategies such as active surveillance in KTRs, as discussed by Mistretta et al.^27^ While the risk of upstaging may be higher in this population, the potential benefits of definitive treatment must be carefully balanced against competing risks such as limited life expectancy also often present in this population.

PSM rates were comparable between KTRs and controls, consistent with prior comparative studies by Felber et al. and Léonard et al.^3^, ^18^ The lone recurrence we observed occurred in a patient with multiple established risk factors, including obesity, pT3a disease, and PSM.^28^, ^29^, ^30^

### 4.4 Graft function

Despite transient postoperative differences in serum creatinine and eGFR between KTRs and matched controls at 30‐days post‐RARP, graft function remained stable long‐term, consistent with prior reports by Léonard et al., Felber et al., and Polcari et al.^3^, ^18^, ^26^

The modest early rise in creatinine (+0.3 mg/dl) and decline in eGFR (−4.5 ml/min/1.73m^2^) likely reflect expected perioperative fluctuations in this immunosuppressed population, rather than clinically meaningful injury. One patient in our series experienced a minor electrolyte disturbance (hyperkalemia) one day after RARP, which was easily manageable. Renal function in KTRs returned to baseline by 6 months, reinforcing the safety of RARP in preserving long‐term graft outcomes when performed by experienced surgeons.

### 4.5 Survival outcomes

While overall mortality was higher in KTRs, the sole death in our cohort was due to graft failure and unrelated to prostate cancer. The association between transplant status and all‐cause mortality seen in both unmatched and IPTW analyses likely reflects the cumulative burden of medical comorbidities inherent to transplant recipients rather than treatment‐related factors. Importantly, no KTR developed metastatic progression or prostate cancer‐specific mortality, supporting the oncologic safety of RARP in this population.

### 4.6 Limitations

This study has limitations. The small sample size of KTRs (*n* = 8) limits statistical power and generalizability, though it remains one of the few studies using a propensity‐matched comparative design. As a single‐system experience, institutional practices may not reflect those in the broader US population. The retrospective design introduces potential selection and information bias. Finally, despite longer follow‐up than many prior reports, the median 39‐months interval may be insufficient to fully assess long‐term oncologic and graft durability in the KTR population with indolent prostatic disease.

## 5 CONCLUSION

In this propensity‐matched cohort study, RARP in KTRs was technically feasible and associated with favourable perioperative, oncologic, and graft‐related outcomes when performed by experienced surgeons. These findings support RARP as a viable treatment option for appropriately selected KTRs, emphasizing the need for individualized surgical planning, close postoperative surveillance, and extended follow‐up in this patient population.

## AUTHOR CONTRIBUTIONS

All authors have approved the final draft and made a significant contribution to the methods and findings in the paper.

## CONFLICT OF INTEREST STATEMENT

The authors declare no conflicts of interest.

## Supporting information


**Table S1:** Perioperative Parameters of RARP Patients by KTR Status.
Table S2: Short and Long‐term Outcomes of RARP Patients by KTR Status.
Table S3: Histopathologic and Oncologic Outcomes of RARP Patients by KTR Status.
Table S4: Overall mortality of Matched Cohort.
